# Risk and contract management practices, and construction projects performance: A test of moderation by managerial skills

**DOI:** 10.1371/journal.pone.0352974

**Published:** 2026-07-07

**Authors:** Waithaka Huria Karugu, Kirema Nkanata Mburugu, Duncan Mugambi Njeru

**Affiliations:** Department of Business Studies, University of Embu, Embu, Kenya; Kwame Nkrumah University of Science and Technology, GHANA

## Abstract

Kenya’s socioeconomic development is heavily dependent on the construction industry; however, poor risk and contract management typically results in cost overruns, delays, and quality deficiencies that undermine project performance. Therefore, this study examined the way risk management practices affect the performance of building projects in the Nairobi Metropolitan Area, considering contract management practices and the moderating effect of project managers’ skills. The Theory of Constraints and Agency Theory, which place a strong emphasis on supervision, responsibility, and the removal of performance barriers, serve as the foundation for the analysis. The study used a descriptive and explanatory research design. 127 completed construction projects (95 residential and 32 non-residential) comprised the target population for this study. From this, 64 valid responses (96% response rate) were obtained from a sample of 67 projects chosen by stratified random sampling using Slovin’s technique at a 95% confidence level. A standardized Likert-scale questionnaire was used to collect data. Inferential statistics, such as simple linear and hierarchical multiple regressions and Pearson’s correlation analysis, were utilized to evaluate relationships and prediction effects. Descriptive statistics, such as means, percentages, and standard deviations, were utilized to define the project’s characteristics. Expert review was used to guarantee the validity of the instrument, and reliability was verified using Cronbach’s alpha coefficients greater than 0.70. The results demonstrate that both risk management practices (β = 0.612, p < 0.001) and contract management practices (β = 0.239, p = 0.020) are significant positive predictors of construction project performance, with the combined model accounting for 60.6% of the variance in construction project outcomes (R² = 0.606). Project manager skills did not significantly moderate the relationship between these management practices and project performance (interaction terms p > 0.05) and exhibited no significant independent effect (β = 0.132, p = 0.120), contributing only a marginal increase in explanatory power to R² = 0.62. The study concludes that while robust risk and contract management frameworks are critical for project success, the additive value of project manager skills remains modest and non-moderative. Recommendations include establishing uniform industry protocols and continued emphasis on professional certification and leadership development to support, rather than substitute systemic management practices.

## 1. Introduction

The construction industry is an important driver of Gross Domestic Product (GDP) growth, employment creation, and infrastructure provision, making it a vital force behind global socioeconomic development. It is predicted that by 2030, the global construction market would have grown to USD 14.8 trillion, or almost 13% of the world’s gross domestic product [1]. In developing economies, the industry contributes up to 14% of the gross domestic product [2]. With more than 100 million workers worldwide, the industry is essential for global economic growth and job creation. Nevertheless, inefficiencies such as cost overruns, delays, and lowered quality plague building projects globally, despite their economic significance. According to a recent global report [3], major construction projects experience substantial overruns, with completion time extensions averaging 67% and costs averaging 33% of the initial budgets.

The significance of risk management practices in enhancing construction project performance is supported by a large body of research. For instance, proactive risk identification, assessment, and mitigation, greatly lower project delays and cost escalations, thereby enhancing performance in Kenya [4]. Quantitative risk analysis and risk response have also been found to influence the performance of construction projects in Kenya [5]. Similarly, a study on construction projects in Pakistan [6], demonstrated that effective risk management practices, such as risk identification, monitoring and prevention, moderated by risk transparency and risk coping capacity, significantly influence construction project success. Contract management practices, such as appropriate contract preparation, administration, and enforcement, have been found to be crucial factors in project success in addition to risk management practices. Research on infrastructure projects in Kenya [7] and internationally [8] highlight that effective contract management practices significantly reduce substandard work, reduce disputes, and enhance project performance.

The function of project manager’s skills is another new area of study in international construction. It has been demonstrated that project success is directly impacted by the technical proficiency, leadership, and soft skills of project managers, including communication and emotional intelligence [9]. Project managers’ competencies have been found to have a moderating effect on the relationship between project risk management and project planning on project success [10]. Kenya’s construction industry has grown rapidly because of government-led infrastructure initiatives under the Big Four Agenda and Vision 2030. According to the Kenya National Bureau of Statistics in 2024, construction output has shown volatility, and the residential building construction subsector has shown some increased growth. However, sector performance remains uneven. Ineffective risk management systems, weak contract management and enforcement, and insufficient project management skills are frequently blamed for delays, cost overruns, and quality deficiencies [8].

The Nairobi Metropolitan Area (NMA), which consists of Nairobi City and the neighboring counties of Kiambu, Machakos, Kajiado and Murang’a, is where these issues are most severe. This area, which serves as the administrative and economic center of Kenya, has seen a boom in public, commercial, and residential construction projects. However, according to a 2024 report by the Auditor General, unexpected hazards due to engagement of unqualified contractors, contract conflicts, and inadequate project monitoring resulted in the abandonment or considerable delay of projects worth over Kenya Shillings 12 billion in this region, among others. Scholarly studies have mostly focused on risk management or contract management separately, despite the region’s significant concentration of building activity. Few studies have examined how project managers’ skills may influence the relationship between risk management practices and project performance, especially in high-risk, high-growth settings such Nairobi Metropolitan area.

There is little empirical information from sub-Saharan Africa, and Kenya specifically, regarding the interactions between risk management, contract management, and project managers’ skills, despite the fact that these characteristics are all key predictors of project success according to international literature [11]. In particular, little is known about how project managers’ skills influence the relationship between risk and contract management practices and project performance. This disparity is crucial because managerial skills may make the difference between a project’s success and failure in situations with fluctuating market conditions, scarce resources and a wide range of stakeholders.

To close this gap, this study examines how risk and contract management practices affect the performance of construction projects in the Nairobi Metropolitan Area considering the moderating role of project manager skills. This study intends to offer theoretical insights by testing the moderating role of project manager’s skills within the integrated framework of Agency Theory and Theory of Constraints. The study offers a more nuanced understanding of how construction project performance is achieved, moving beyond the direct effect models.

### 1.1. Theoretical framework

The theoretical underpinnings of this study include Agency Theory and the Theory of Constraints (ToC), which offer a framework for relating construction project performance to risk management practices, contract management practices, project manager skills, and so on. Agency Theory explains the interaction between project owners (principals) and contractors or project managers (agents) [12]. According to this idea, agents may behave in their own self-interest, which could lead to hazards such as delays, cost overruns, or degraded quality because of information asymmetry and competing goals. In this context, risk management practices (independent variables) serve as mechanisms to minimize agency problems by strategically and systematically identifying, monitoring, and mitigating risks that emerge from the principal-agent relationship. Similarly, contract management practices (independent variable) reduce agency costs by ensuring compliance with agreed-upon timelines, budgets, and quality standards, thus aligning the interests of both parties [13]. Furthermore, this theory places a high value on the project manager’s abilities (moderating variable), since managerial skills in communication, negotiation, and monitoring guarantee accountability, curb opportunistic behavior, and enhance overall project performance (dependent variable).

This perspective is supported by the Theory of constraints (ToC) developed by Eliyahu M. Goldratt in the year 1984 which highlights how a limited number of restrictions or inefficiencies frequently limit the performance of an organization. The limitations of construction projects include tight deadlines, restricted funds, and a lack of technical expertise. The ToC asserts that good risk management practices aid in locating and resolving obstacles before they cause the project failure, thereby enhancing construction project performance [14]. Crucially, ToC emphasizes the role of project managers’ abilities as a moderating factor: highly skilled managers are better able to anticipate, prioritize, and resolve constraints, thereby amplifying the positive effect of risk management practices on project performance. Similarly, contract management practices are crucial for strengthening weak links by ensuring that roles, responsibilities, and deliverables are clearly defined and enforced [8].

Therefore, while ToC identifies bottlenecks in construction project workflows and emphasizes the significance of resolving systemic constraints through competent project management, Agency Theory explains information asymmetry and incentive misalignment in construction project contracts and supports the inclusion of risk management and contract management practices in this study as a means of aligning stakeholder interests. Combining ToC and Agency Theory will allow modelling of how the Agency Theory explains the contractual dynamics of the contracting parties, while ToC explains systemic bottlenecks in a construction project flow. Therefore, ToC will be used to identify where agency problems (risks) manifest in construction projects in Kenya, while contextualizing the boundaries of the two theories.

## 2. Methodology

### 2.1. Study Area

Nairobi County and portions of Kiambu, Kajiado, Murang’a, and Machakos counties comprise the Nairobi Metropolitan Area (NMA), where this study was conducted in Kenya. The NMA, which covers an area of over 30,000 square kilometers and is the commercial and infrastructure center of the nation, is distinguished by its high population density, rapid urbanization, and thriving real estate market. Real estate, manufacturing, services, and commerce power its economy, with construction being a key driver of employment and economic growth. The area was specifically chosen because of the large number of completed and ongoing construction projects there, as well as the variety of parties actively involved in the industry, including developers, architects, contractors, project managers, engineers, and quantity surveyors. The study’s target respondents were professionals actively involved in project execution, and it focused on 127 completed residential and non-residential projects. It was perfect to examine how risk management, contract management, and project managers’ skills and abilities affect project performance by concentrating on this vibrant and rapidly expanding metropolitan area, where issues such as cost overruns, delays, quality deficiencies, and contract disputes are frequent.

### 2.2. Study variables

Numerous studies view project success as a multifaceted construct evaluated based on client satisfaction, quality, cost, and time. Stakeholder satisfaction and strategic alignment are as important to project success as meeting budgets and schedules [15]. Similarly, it has been noted that traditional parameters for measuring project performance such as punctuality, cost control, and quality delivery, are still dominant, and there is a need to use more modern performance yardsticks to measure project performance [16]. This study uses risk and contract management practices as the independent variables, project manager’s skills as a moderating variable and project performance as the dependent variable. Table 1 shows the study variables and how Likert-scale ratings were employed in this study to operationalize project performance.

**Table 1:** Study variables description.

Variable Category	Variables	Description	Measurement Approach
Dependent Variable	Construction Project Performance	The overall success of construction projects is determined by timeliness, cost control, quality compliance, and client satisfaction.	Measured using a 5-point Likert scale (1 = Strongly Disagree to 5 = Strongly Agree); a composite score was computed as the average of the six items, retaining the original 1–5 continuous scale. Higher scores indicate better performance..
Independent Variable	Risk Management Practices	Systematic identification, analysis, monitoring, and mitigation of risks in construction projects.	The items were measured on a 5-point Likert scale (1 = Strongly Disagree to 5 = Strongly Agree), and a composite score was computed as the average of the six items, retaining the original 1–5 continuous scale. Higher scores indicate more extensive risk management.
Independent Variable	Contract Management Practices	Processes and procedures in planning, executing, and administering contracts to ensure compliance with cost, time, and quality requirements.	Measured on a 5-point Likert scale (1 = Strongly Disagree to 5 = Strongly Agree), a composite score was computed as the average of the four items, retaining the original 1–5 continuous scale. Higher scores indicate more effective contract- management practices.
Moderating Variable	Project Manager’s Skills	Technical and soft skills of project managers, including leadership, decision-making, problem-solving, communication, and emotional intelligence.	The items were measured on a 5-point Likert scale (1 = Strongly Disagree to 5 = Strongly Agree), and a composite score was computed as the average of the six items, retaining the original 1–5 continuous scale. Higher scores indicate a greater perceived importance of these skills.

Improved project outcomes have been repeatedly associated with risk management practices, including risk identification, assessment, monitoring and mitigation. Strong risk identification and reporting improved project completion at the Kenya Airports Authority [17]. Brainstorming and risk assessment have been highlighted as crucial tactics for borehole projects in Mombasa [18]. Similarly, past studies showed that risk management considerably influenced the association between planning and project success in both Pakistan and the UK [19]. Stakeholder views on how well risks are handled throughout building projects are used in this study to gauge risk management practices as an independent variable.

It is often known that in construction projects, contract management influences how well the project performs. While some research show that contract monitoring and documentation significantly impacted project delivery in Nigeria [20], others demonstrated that contract cost management and control enhanced the performance of county projects in Kenya [21]. Effective project outcomes in Zambia depend on adherence to pre- and post-award agreements [22]. Consistent with these conclusions, this study uses contract management practices as an independent variable, acknowledging that, similar to risk management practices, they have a notable impact on project performance.

It has been emphasized in several situations that project managers are the central decision-makers responsible for analyzing, interpreting, implementing and adapting project management frameworks in construction projects [23]. Project manager’s skills have been found to significantly moderate the relationship between project success and other factors such as organizational culture and communication management, with project managers who are competent being able to prevent negative influences on project outcomes [24]. Soft skills such as leadership, communication, and emotional intelligence, improve performance results in challenging projects. Research shows that project managers with higher emotional intelligent, possess strong leadership skills and the capability to lead a project to successful completion and achieve better project outcomes [25]. This study uses the skills of the project manager as a moderating variable to test its contribution to enhancing the beneficial impact of risk and contract management practices on the performance of construction projects.

### 2.3 Research design, sample size, and sampling strategy

This study used a research strategy that was both descriptive and causal (explanatory). A descriptive research design was used to provide a thorough description of the current state of risk management practices, contract management, project managers ‘skills, and project performance in the Nairobi Metropolitan Area. Using this methodology, the study was able to gather quantifiable information on the attitudes and behaviors of stakeholders, providing a thorough grasp of the current situation of construction project management today [26]. A causal-effect research design was used to determine the cause-and-effect relationship between the study variables. In particular, the design made it possible to assess the relationship between risk and contract management practices (independent variables) and construction project performance (dependent variable), while also analyzing the moderating effect of managerial skills. By incorporating this methodology, this study examined the predictive ability of risk and contract management practices on project performance and whether project managers’ skills moderate these relationships.

The breadth and depth of the analysis were guaranteed by the combination of explanatory and descriptive study designs. Descriptive statistics provided an overview of the characteristics and distribution of variables across the sample. Explanatory analysis through simple linear and hierarchical multiple regression models was employed to test the statistical effects of risk and contract management practices on project performance, as well as the moderating role of the project manager’s skills. These designs were chosen to address the study objectives and provide empirical evidence about the impact of project risk and contract management practices, moderated by managerial skills, on construction project performance in a high-risk and dynamic construction project environment. This study focused on 127 completed construction projects; 95 residential and 32 non-residential in the Nairobi Metropolitan Area. Slovin’s method, frequently used in business and management research and has been utilized in studies related to construction projects [27], was applied at a 95% confidence level to determine a representative sample. The sample size was calculated using Equation 1.


$$\begin{array}{c} \text{n}=\text{N}/(\text{1}+{\text{Ne}}^{2}) \end{array}$$ (1)

where n is the sample size, N is the total population (127), and e is the margin of error (0.05 margin of error). Therefore, the sample size was 96. To ensure the representation of both project categories and maintain methodological rigor, stratified sampling was performed, yielding a sample of 67 projects: 50 residential and 17 non-residential (Table 2), from which responses from 64 respondents were received representing a response rate of 96%. This sample provides adequate statistical power for linear regression based on Jenkins and Quintana-Ascencio [28], who recommended a sample size N ≥ 25. In addition, a power analysis [29] indicates that with N = 64, α = 0.05 and three predictor variables, the study’s power to detect medium effect sizes (f^2^ = 0.15) is > 0.80, the conventional allowable threshold. Therefore, the sample size was adequate for detecting medium to large effect sizes but may be underpowered for detecting small effect sizes.

**Table 2:** Sample selection.

Project Type	Population	Sample	Percentage
Residential Construction Projects	95	50	75%
Non-Residential Construction Projects	32	17	25%
**Total**	**127**	**67**	**100%**

The reliability, validity, and generalizability of the study findings were improved by this strategy, which ensured that both residential and non-residential construction projects were fairly represented. To guarantee a proportionate representation of both residential and non-residential construction projects in the Nairobi Metropolitan Area, the study used a stratified random selection technique.

### 2.4 Ethical consideration

This study was conducted in compliance with the established research ethics. Approval to conduct the research was given by the University of Embu. Participation was voluntary, and informed consent was obtained from all the respondents. Consent was obtained in written form by asking the participants to confirm their consent through the question *“Do you consent to your responses being used for this research purposes only?”* Their responses would be used solely for the study. Anonymity and confidentiality were assured, and the data were used solely for academic purposes. Prior to collecting field data, a research permit License Number (***NACOSTI/P/25/416014)***, was acquired from The National Commission for Science, Technology and Innovation (NACOSTI). The researchers declare no conflicts of interest, and the findings are presented objectively and responsibly.

### 2.5 Data collection instrument

A structured questionnaire was created based on earlier empirical research in project management and customized to the study’s goals to gather data. The questionnaire comprised five sections: (i) respondent demographics, (ii) risk management practices, (iii) contract management practices, (iv) project managers ‘skills, and (v) construction project performance. A five-point Likert scale, with 1 denoting “strongly disagree” and 5 denoting “strongly agree,” was used to quantify all study variables. This allowed respondents to express their level of agreement with statements about project techniques and results. This scaling method is frequently employed in management research because it can standardize and quantify perceptions and attitudes [30]. Before administration, the validity and reliability of the questionnaire were examined. Expert review was used to ensure content validity, and Cronbach’s alpha was used to test internal consistency. All constructs met the acceptable reliability standards (α > 0.70). The Likert scale made statistical analysis using descriptive and inferential approaches easier, and the instrument’s organized nature improved uniformity among responses.

### 2.6 Statistical analysis

Using SPSS 23.0, the data gathered from the structured questionnaires were coded, cleaned, and examined. According to the goals of this study, the analysis included both descriptive and inferential statistics. The key characteristics of the study variables- risk management practices, contract management practices, project managers ‘skills, and construction project performance- were first summed up using descriptive statistics such as means, standard deviations, percentages, and frequency distributions. These statistics provide an overview of the degree of management practice implementation and perceived project outcomes across the sampled projects. Two academic experts and three construction project management experts were consulted to assess the validity of the research instrument. Each expert independently assessed the questionnaire for clarity and relevance. Their feedback was collated systematically; the ambiguous items were revised for clarity and redundant questions were removed. Additionally, the consistency and relevance of the constructs were examined using correlation analysis utilizing Pearson’s correlation coefficients [31], and the items’ conformity with previous empirical research was used to establish content validity. The Cronbach’s alpha coefficient was also employed to evaluate the reliability of the questionnaire; all constructs were found to have good internal consistency, above the suggested threshold of 0.70, as shown in Table 3.

**Table 3:** Reliability results table.

Variable	Number of Items	Cronbach’s Alpha
Risk Management Practices (RMP)	6	0.803
Contract Management Practices (CMP)	4	0.872
Project Manager’s Skills (PMS)	6	0.838
Project Performance (PP)	6	0.909

A multiple linear regression model was adopted as the main analytical tool to examine the effect of risk and contract management practices on construction project performance and the moderating effect of managerial skills. This model was considered suitable because project performance was operationalized as a continuous composite score, obtained by averaging the measurement items of quality, time, client’s satisfaction, safety, budget and environment, which were each measured on a Likert scale of 1–5. Risk and contract management practices were also computed as composite scores. To test the moderating effect of project manager’s skills a multiple linear regression model was used with the introduction of interaction terms.

The models that examined the effect of risk and contract management practices on project performance are as indicated in Equations 2 and 3 below.


$$\begin{array}{c} PP=\beta_{\text{0}}+\beta_{\text{1}}RMP+\varepsilon \end{array}$$ (2)

$$\begin{array}{c} PP=\beta_{\text{0}}+\beta_{\text{1}}CMP+\varepsilon \end{array}$$ (3)

Where:

*PP* is Construction Project Performance

*RMP* is Risk Management Practices

CMP is Contract Management Practices

β_0_ is Intercept

β_1_ is Regression coefficient

Moderation Model is as indicated in equation 4:


$$\begin{array}{c} PP=\beta_{\text{0}}+\beta_{\text{1}}RMPc+\beta_{\text{2}}CMPc+\beta_{\text{3}}PMSc+\beta_{\text{4}}(RMPc\times PMSc)+\beta_{\text{5}}(CMPc\times PMSc)+\varepsilon \end{array}$$ (4)

Where: RMPc,CMPc,PMSc  are Centred variables RMPc×PMSc is the Interaction between risk management and project manager’s skills CMPc×PMSc is the Interaction between contract management and project manager’s skills

The significance of the regression coefficients was evaluated using p-values at 0.05 significance level. Model fit was assessed using R-squared (R²) and F-statistics. Additionally, multicollinearity diagnostics were performed using Variance Inflation Factor (VIF), where values below 10 indicated no multicollinearity concerns. This statistical framework was suitable since it addressed the main goals of the study by enabling the simultaneous evaluation of the conditional (moderating) effect of project managers’ abilities as well as the direct effects of risk management and contract management practices on project performance.

## 3. Results and discussion

### 3.1 Descriptive characteristics of the study

The Nairobi Metropolitan Area’s building projects showed a moderate to high application of management methods and performance outcomes, according to the descriptive data in Table 4. A mean rating of 4.08 (SD = 0.35) for risk management practices indicates that the majority of projects used methodical techniques for risk identification, analysis, and mitigation. This result is in line with that of Mutunga and Ondara [17]) who found that the Kenya Airports Authority greatly decreased delays and cost overruns through efficient risk identification and reporting. In a comparable manner, it was discovered that organized risk assessment techniques, like pilot testing and brainstorming, improved the success of borehole projects in Kenya [18]. Mbogori and Mutuku [32] found that risk management have positive and significant effect on construction project performance. The idea that proactive risk management is becoming more and more institutionalized in Kenyan building projects is supported by these studies taken together.

**Table 4:** Descriptive statistics.

Variable	No of observations	Mean	St.dev	Min	Max
Risk management practices	64	4.08	0.35	3.00	5.00
Contract management practices	64	4.09	0.32	3.00	5.00
Project managers’ skills	64	4.55	0.41	3.00	5.00
Project performance	64	4.17	0.37	3.00	5.00

At a mean of 4.09 (SD = 0.32), is an indication that contract management practices are effectively implemented in construction projects. This is consistent with Mwangi [33], who found that contract cost control and management had a favorable impact on Kiambu County project delivery. The results indicate that contract management practices remain an essential supporting framework in construction projects. With a mean score of 4.55 (SD = 0.41) (Table 4), project managers’ skills were the most highly evaluated variable, suggesting that managerial skills were thought to have a significant impact on project results. This corroborates the findings of Rodrigues and Matos [34], who showed that the leadership abilities and emotional intelligence of project managers were important indicators of performance in large-scale projects. In the same way, Fazly et al. [10] verified that the efficacy project performance in Afghanistan’s construction industry was improved by the interpersonal and leadership abilities of project managers. The study’s high evaluation of managerial abilities emphasizes how important human elements are in construction projects.

With a mean score of 4.17 (SD = 0.37), the construction project performance itself showed comparatively positive results in terms of punctuality, cost control, safety, client’s satisfaction and quality and environmental compliance. Jabongo [35] found that result based project monitoring in construction projects is positively associated with project performance across several dimensions, such as those in this study, which is consistent with this finding. In a comparable way, Siddiqui et al. [36] contended that project success goes beyond fulfilling financial and schedule obligations, emphasizing the significance of stakeholder satisfaction, which was demonstrated by the favorable opinions documented in this study.

In conclusion, the descriptive results are consistent with earlier studies that highlight the role that managerial skills, contract management practices and risk management practices have in influencing construction project performance.

### 3.2 Correlation analysis of study variables

To investigate the direction and strength of relationships between the main research variables; risk management practices, contract management practices, project managers ‘skills, and construction project performance, correlation analysis was performed (Table 5).

**Table 5:** Correlation analysis.

Variables	RMP	CMP	PMS	PP
Risk Management (RMP)	1.000	0.597**	0.301*	0.755**
Contract Management (CMP)	0.597**	1.000	0.088	0.604**
Project Manager Skills (PMS)	0.301*	0.088	1.000	0.324**
Project Performance (PP)	0.755**	0.604**	0.324**	1.000

**Note:**

** Correlation is significant at the 0.01 level (2-tailed)* Correlation is significant at the 0.05 level (2-tailed)

RMP, risk management practices; CMP, contract management practices; PMS, project manager’s skills; PP, project performance

Higher levels of construction project performance in the Nairobi Metropolitan Area are continuously linked to changes in project management practices and managerial competencies, according to the results, which showed statistically significant positive connections across all variables. Project performance showed a strong positive and statistically significant relationship link with risk management practices (r = 0.755, p < 0.01). This suggests that projects using a systematic approach to risk identification, assessment, and mitigation had a higher chance of successful project performance. This result is in line with that of Mutunga and Ondara [17], who found that the Kenya Airports Authority reduced delays and cost overruns by thorough risk identification and reporting. In agreement with the findings, Song et al. [37] found that risk management practices such as risk identification, assessment and mitigation enhance sustainable project performance, while a study by Yacila and Johnson [38], found that effective risk management practices has a direct correlation with improved project performance with regards to adherence to project timelines, quality and cost control. These findings demonstrate how structured risk management offers protection against unforeseen circumstances and has a direct impact on construction project delivery results.

Additionally, there was a favorable correlation between contract management practices and project performance (r = 0.604, p < 0.01). This emphasizes how important it is to have a clear contract design, cost control procedures, and project monitoring systems to make sure that building projects stay on schedule and meet quality requirements. This conclusion is supported by empirical data from Mwangi [33] in Kiambu County, which demonstrates that contract management and contract cost control were important factors in determining the success of county government projects. Similarly, Alshboul and Shehadeh [39] demonstrated that optimized contract structures and well-defined contract clauses reduce poor performance factors in construction projects such as project delays. Shi et al. [40] found that strategic contract management practices align the incentives and obligations of the parties to a construction contract and thereby enhancing timely project delivery and successful performance. The study’s substantial association highlights how crucial it is to institutionalize contract management procedures as a major factor in project success.

Project managers’ skills and project performance showed the significant correlation (r = 0.324, p < 0.01), indicating that managerial skills like communication, leadership, decision-making, and emotional intelligence are essential for a project’s successful completion. Dastgeer et al. [41] and Shin and Kang [42] found that project managers with relevant competencies enhanced performance of construction projects, both time and quality through enhanced construction team performance and better process management in construction projects. The Nairobi context’s high correlation indicates that human factors continue to play a critical role in determining the success or failure of infrastructural initiatives.

From the analysis it is also revealed that risk management practices and contract management practices are positively and significantly related (r = 0.597, p < 0.01), indicating that projects with strong risk management systems are likely to also exhibit effective contract management practices. Further analysis revealed a weak but statistically significant (r = 0.301, p < 0.05) relationship between risk management practices and project manager’s skills, an indication of a slight association between managerial competencies and the implementation of risk management practices. The relationship between contract management practices and project manager’s skills was weak and not statistically significant (r = 0.088, p > 0.05), indicating no meaningful association between these variables. On overall, the analysis indicates a strong significant relationship between the independent variables and project performance, hence further regression analysis to determine the extent to which the independent variables influence construction project performance.

### 3.3 Regression results on project performance

#### 3.3.1 Effect of Risk Management Practices on Construction Project Performance.

The following model was used:


$$\begin{array}{c} PP=\beta_{\text{0}}+\beta_{\text{1}}RMP+\varepsilon \end{array}$$ (2)

Where:PP is Project Performance, RMP is Risk Management Practices, $\beta_{\text{0}}$ is Intercept, $\beta_{\text{1}}$ is Regression coefficient and ε is Error term

From the regression model, a statistical significance (p < 0.001) was demonstrated, confirming that risk management practices are a meaningful predictor of performance of construction projects. Based on the model results, the coefficient of determination (R² = 0.570) suggests 57% of the variance on the outcomes of construction project performance can be attributed to risk management practices. The analysis further yielded a positive and statistically significant coefficient for risk management (β = 0.755, p < 0.001), an indication that enhanced risk management practices correspond to notable improvements in performance of construction projects (Table 6). These results align with previous research by Isack & Chege [43] and Cherono & Nyang’au [44], who similarly documented that effective risk management enhances construction project success across diverse contexts.

**Table 6:** Regression Results.

Variable	B	Std. Error	Beta	t	p-value
**Regression Results (Risk Management → Project Performance, R² = 0.570, p < 0.001)**					
Constant	1.343	0.475	—	2.828	0.006
Risk Management	0.961	0.106	0.755	9.058	0.000
**Regression Results (Contract Management → Project Performance, (R² = 0.365, p < 0.001)**					
Constant	1.343	0.475	—	2.828	0.006
Contract Management	0.692	0.116	0.604	5.973	0.000
**Multiple Regression Results (RMP & CMP → Project Performance, F(2,61) = 46.968, p < 0.001, R² = 0.606, Adj. R² = 0.593)**					
Constant	−0.126	0.447	—	−0.282	0.779
Risk Management	0.779	0.127	0.612	6.111	0.000
Contract Management	0.273	0.115	0.239	2.385	0.020

B, unstandardized regression coefficient; Std error, standard error; Beta, standardized regression coefficient; t, t-statistic for testing the predictors; p, significance level; R^2^, coefficient of significance for the predictors; Adj R^2^, adjusted R^2^, F, F-statistic for the regression model

#### 3.3.2 Effect of Contract Management Practices on Construction Project Performance.

The following model was used:


$$\begin{array}{c} PP=\beta_{\text{0}}+\beta_{\text{1}}CMP+\varepsilon \end{array}$$ (3)

Where:PP is Project Performance, CMP is Contract Management Practices, $\beta_{\text{0}}$ is Intercept, $\beta_{\text{1}}$ is Regression coefficient and ε is Error term.

The regression findings, which indicate that the model achieved statistical significance (p < 0.001), confirming that contract management practices are a meaningful predictor of construction project performance. With an R² value of 0.365 it can be observed that contract management practices explain approximately 36.5% of the observed variation in construction project performance. The regression coefficient for contract management was both positive and statistically significant (β = 0.604, p < 0.001), demonstrating that enhanced contract management practices contribute to superior project performance outcomes. (Table 6). Previous similar studies by Kithinji et al. [45], have also identified a positive association between contract management effectiveness and project performance metrics.

#### 3.3.3 Combined Effect of Risk and Contract Management Practices on Project Performance.

The following model was used:


$$\begin{array}{c} PP=\beta_{\text{0}}+\beta_{\text{1}}RMP+\beta_{\text{2}}CMP+\varepsilon \end{array}$$ (5)

Where:

PP represents construction projects performance, $\beta_{\text{0}}$ is a constant term, $\beta_{\text{1}}RMP$ is the regression coefficient for risk management practices, $\beta_{\text{2}}CMP$ is the regression coefficient for contract management practices, ε is the error term.

The overall model exhibited statistical significance (F (2,61) = 46.968, p < 0.001), indicating that the predictor variables collectively account for variability in construction project performance. The analysis produced an R² value of 0.606, suggesting that risk management and contract management practices together explain approximately 60.6% of the variance in construction project performance. Both managerial practices demonstrated a positive and statistically significant contribution to construction project performance, with (β = 0.612, p < 0.001) for risk management practices and (β = 0.239, p = 0.020) for contract management practices. From these findings it can be demonstrated that while both managerial practices are significant determinants of construction project performance, risk management practices show a comparatively stronger influence that contract management practices.

#### 3.3.4 Multicollinearity Diagnostics.

To evaluate potential multicollinearity among the predictor variables, diagnostics assessments were conducted. Variance Inflation Factor (VIF) values for both risk management and contract management practices were 1.554, substantially below the conventional threshold of 10. Furthermore, tolerance values exceeded 0.1, confirming the absence of problematic multicollinearity within the model. These diagnostic results support the reliability and interpretability of the regression estimates, (Table 7)

**Table 7:** Multicollinearity Diagnostics.

Variable	Tolerance	VIF
Risk Management	0.643	1.554
Contract Management	0.643	1.554

#### 3.3.5 Moderation Effect of Project Managers Skills on Relationship Between Risk & Contract Management Practices and Project Performance.

A hierarchical multiple regression analysis was performed to evaluate whether project manager’s skills moderate the associations between risk management practices, contract management practices, and construction project performance. All predictor variables were mean-centered prior to analysis to reduce potential multicollinearity and to facilitate clearer interpretation of interaction effects.

In the first step, the centered independent variables (risk management and contract management) along with the proposed moderator (project manager’s skills) were entered into the model. This model was specified as:


$$\begin{array}{c} PP=\beta_{\text{0}}+\beta_{\text{1}}RMPc+\beta_{\text{2}}CMPc+\beta_{\text{3}}PMSc+\varepsilon \end{array}$$ (6)

Where:

PP= Project Performance, ${RMP}_{C}$ = Centered Risk Management Practices, ${CMP}_{C}$ = Centered Contract Management Practices, ${PMS}_{C}$ = Centered Project Manager’s Skills and ε = Error term

The overall model demonstrated statistical significance (F(3,60) = 32.902, p < 0.001) and accounted for 62.2% of the variance in project performance (R² = 0.622) (Table 8). Within this initial model, risk management practices emerged as a significant positive predictor (β = 0.561, p < 0.001), and contract management practices also retained significance (β = 0.258, p = 0.012) (Table 8). However, project manager’s skills did not achieve statistical significance (β = 0.132, p = 0.120) (Table 8).

**Table 8:** Regression model results.

Variable	Beta	t	p-value
**Main Effects Model – (R² = 0.622, Adj. R² = 0.603, F (3,60) = 32.902, p < 0.001)**			
Risk Management (Centered)	0.561	5.387	<0.001
Contract Management (Centered)	0.258	2.586	0.012
Project Manager Skills (PMS_c)	0.132	1.576	0.120
**Moderation Model (Interaction Effects) – (R² = 0.629, Adj. R² = 0.598, F (5,58) = 19.706, p < 0.001)**			
Risk Management (Centered)	0.558	4.828	<0.0001
Contract Management (Centered)	0.273	2.694	0.009
Project Manager Skills (PMS_C)	0.122	1.395	0.168
RMP × PMS	−0.062	−0.581	0.563
CMP × PMS	0.105	1.085	0.283

Beta, standardized regression coefficient; t, t-statistic for testing the significance of predictors; p, probability value; R^2^, coefficient of determination; Adj R^2^, adjusted R^2^, F, F-statistic for the regression model

In the second step, the interaction terms (RMPc × PMSc and CMPc × PMSc) were introduced to test for moderation effects. The expanded model is expressed as:


$$\begin{array}{c} PP=\beta_{\text{0}}+\beta_{\text{1}}RMPc+\beta_{\text{2}}CMPc+\beta_{\text{3}}PMSc+\beta_{\text{4}}(RMPc\times PMSc)+\beta_{\text{5}}(CMPc\times PMSc)+\varepsilon \end{array}$$

Where: RMPc,CMPc,PMSc  = Centered variables, RMPc×PMSc= Interaction between risk management and skills, CMPc×PMSc = Interaction between contract management and skills and ε *=* Error term

The addition of the interaction terms yielded a marginal increase in explanatory power, with R² rising slightly from 0.622 to 0.629. The model remained statistically significant (F(5,58) = 19.706, p < 0.001). Nevertheless, both interaction effects were found to be non-significant: RMP × PMS (β = −0.062, p = 0.563) and CMP × PMS (β = 0.105, p = 0.283) (Table 8). The minimal improvement in R² further indicates that incorporating the interaction terms provided negligible additional explanation of construction project performance variance. These results indicate that project manager’s skills do not meaningfully moderate the relationships between risk management practices, contract management practices, and construction project performance. While project manager’s skills may be considered valuable in absolute terms, they do not appear to amplify or diminish the effects of formal risk and contract management practices on project outcomes.

The descriptive analysis revealed that project manager’s skills were perceived as highly important by respondents; however, the regression findings indicate that these competencies do not appreciably strengthen or diminish the performance effects of risk and contract management systems. This pattern implies that structured organizational frameworks for risk and contract management may play a more dominant role in shaping project outcomes than individual managerial attributes. These demonstrate that formalized management practices establish robust foundations for project execution, while project manager competencies contribute independently rather than interactively. The findings suggest that management practices and project manager skills influence project outcomes through independent, additive mechanisms rather than through interactive or conditional relationships.

The results are theoretically consistent with the Agency Theory, which holds that opportunism and knowledge asymmetry between project owners (principals) and contractors (agents) are lessened by efficient monitoring through risk and contract management. These methods save agency expenses and improve project results by using accountability procedures. The findings also support the Theory of Constraints (ToC), which highlights how project performance may be greatly enhanced by locating and resolving major bottlenecks, such as risks, contractual gaps, or resource limitations. In conclusion, the regression analysis demonstrates that contract management and risk management practices are important and effective indicators of the success of building projects. These findings highlight the need to improve organizational capability in risk and contract management if the Nairobi construction industry is to satisfy the needs of Kenya’s Vision 2030 infrastructure plan and the country’s fast urban growth.

## 4. Conclusion and policy recommendations

The purpose of this study was to investigate how the performance of construction projects in the Nairobi Metropolitan Area is impacted by risk management practices, contract management practices, and the moderating effect of project managers’ skills. The findings confirm that structured management frameworks are essential for lowering cost overruns, avoiding delays, and guaranteeing adherence to quality standards, thereby enhancing construction project performance. They also show that risk and contract management practices greatly increase the probability of project success. Additionally, the results showed that the relationship between risk and contract management and project performance is not moderated by the skills of project managers. However, high levels of technical, leadership, and interpersonal competences were found to directly enhance the project performance, independent of risk and contract management practices.

These findings theoretically support the applicability of the Agency Theory, which highlights the value of supervision and contract enforcement in reducing agency costs and balancing the interests of contractors and project owners. Similar to this, the Theory of Constraints (ToC) has been proven to work when competent managers find and fix bottlenecks in risk management, schedules, and resource allocation. In general, this study shows that the effectiveness of construction projects in the Nairobi Metropolitan Area depends crucially on the skills of project managers in addition to strong management practices. The framework for success is provided by efficient risk and contract management, while project manager’s skills independently predict success without moderating the effect of the managerial practices. The study’s findings suggest a boundary condition on the application of the Agency Theory, in that while the theory has focused in information asymmetry between the Client’s and Contractors, project manager’s skills may not reduce the agency costs associated with contract and risk management practices, which are already codified in formalized systems. The findings challenge the Theory of Constraints by suggesting that bottlenecks lie in the system designs rather than human expertise.

Given that risk management practices showed a strong positive effect on performance of construction projects, the study recommends that industry practitioners adopt risk management frameworks that are aligned to ISO 31000 in construction projects and ensure that there are regular risk register audits throughout the lifecycle of a construction project. Implementation of such frameworks should be made mandatory and enforced through regulators such as the National Construction Authority (NCA).

The study found contract management practices to have a positive and significant effect on project performance, and the effect does not depend on project manager’s skills. The study therefore recommends that industry practitioners and regulators develop a standard contract compliance indicators and automated contract management software, and embedment of such contract compliance indicators into the project dashboard, whose monitoring should not be left solely at the discretion of the project manager.

Simultaneously, deliberate investment in project managers’ professional development through accreditation, certification, and ongoing training that combines technical and leadership skills will guarantee that management techniques are not just planned but also successfully implemented. When combined, these actions put Kenya’s construction industry in a position to provide robust, effective projects that support the nation’s Vision 2030 development strategy.

Since the study was cross-sectional, future studies should consider longitudinal designs to examine how the managerial practices evolve over different phases of a construction project. In addition, the study’s findings were limited to residential and nonresidential projects in Kenya’s Nairobi Metropolitan Area, which may limit generalizability and therefore future studies should consider comparative studies across other metropolitan areas and countries in the region.

## Supporting information

S1 Data Raw data on the study influence of risk and contract management practices and project manager's skills on performance of construction projects. (PDF)
