# Supplementary material for: Risk and contract management practices, and construction projects performance: A test of moderation by managerial skills
Source: PLoS One. 2026 Jul 7;21(7):e0352974. doi: 10.1371/journal.pone.0352974 (PMC13340765; doi:10.1371/journal.pone.0352974)
Supplement: S1 Data — (PDF) [file pone.0352974.s001.pdf]

RAW DATA ON THE STUDY INFLUENCE OF RISK AND CONTRACT MANAGEMENT PRACTICES AND PROJECT MANAGER'S SKILLS ON PERFORMANCE OF CONSTRUCTION PROJECTS

RISK MANAGEMENT PRACTICES

Indicate the extent to which you agree or disagree with the following statements on risk management practices. Use a scale of 1-5 where 1=strongly disagree, 2=disagree, 3- neutral, 4= agree and 5= strongly agree.

Q1 Construction projects I have been involved in have a formal process for identification potential risks.

Q2 Regular assessments are conducted to evaluate the impact and likelihood of identified risks

Q3 Projects I have been involved in have a comprehensive risk responses plan

Q4 Resources are allocated for implementation of risk responses

Q5 Risk management strategies are regularly monitored and reviewed

Q6 Risk management strategies are adjusted should new risks occur during project implementation

Response ID	Q1. Process	Q2.Assess	Q3. Plan	Q4. Resources	Q5. Monitor	Q6. Adjust
1	5	5	4	4	5	4
2	4	4	4	4	5	5
3	4	4	4	3	4	4
4	5	5	5	4	5	5
5	4	4	4	4	4	4
6	4	4	3	3	4	4
7	4	3	4	3	4	4
8	4	4	4	4	5	4
9	5	5	4	4	5	4
10	4	4	4	4	4	4
11	4	4	4	4	5	4
12	4	4	4	4	4	4
13	4	4	4	4	4	4
14	4	4	4	3	4	4
15	4	4	4	4	5	5
16	4	4	4	4	4	4
17	4	4	4	4	4	4
18	5	4	4	4	5	4
19	4	4	4	4	4	4
20	4	4	4	3	4	4
21	4	4	4	4	5	5
22	5	5	5	4	5	5
23	4	4	4	4	4	4
24	4	4	3	3	4	4
25	4	4	4	4	4	4
26	4	4	4	4	4	4
27	4	4	4	4	5	4
28	4	4	4	3	4	4
29	4	4	4	4	5	4
30	4	4	4	4	4	4
31	4	4	4	4	4	4
32	4	4	4	4	5	5
33	4	4	4	4	4	4
34	4	4	4	4	4	4

RAW DATA ON THE STUDY INFLUENCE OF RISK AND CONTRACT MANAGEMENT PRACTICES AND PROJECT MANAGER'S SKILLS ON PERFORMANCE OF CONSTRUCTION PROJECTS

RISK MANAGEMENT PRACTICES

Indicate the extent to which you agree or disagree with the following statements on risk management practices. Use a scale of 1-5 where 1=strongly disagree, 2=disagree, 3- neutral, 4= agree and 5= strongly agree.

Q1 Construction projects I have been involved in have a formal process for identification potential risks.

Q2 Regular assessments are conducted to evaluate the impact and likelihood of identified risks

Q3 Projects I have been involved in have a comprehensive risk responses plan

Q4 Resources are allocated for implementation of risk responses

Q5 Risk management strategies are regularly monitored and reviewed

Q6 Risk management strategies are adjusted should new risks occur during project implementation

Response ID	Q1. Process	Q2.Assess	Q3. Plan	Q4. Resources	Q5. Monitor	Q6. Adjust
35	4	4	4	4	4	4
36	5	5	4	4	5	5
37	4	4	4	4	4	4
38	4	4	4	4	4	4
39	4	4	4	4	5	4
40	4	4	4	4	4	4
41	4	4	4	4	4	4
42	4	4	4	3	4	4
43	4	4	4	4	5	4
44	4	4	4	4	4	4
45	4	4	4	4	4	4
46	4	4	4	4	4	4
47	4	4	4	4	4	4
48	4	4	4	4	5	4
49	4	4	4	4	4	4
50	4	4	4	4	4	4
51	4	4	4	4	4	4
52	4	4	4	4	4	4
53	4	4	4	4	5	4
54	4	4	4	4	4	4
55	4	4	4	4	4	4
56	5	5	5	4	5	5
57	4	4	4	4	4	4
58	4	4	4	4	4	4
59	4	4	4	3	4	4
60	4	4	4	4	4	4
61	4	4	4	4	5	4
62	4	4	4	4	4	4
63	4	4	4	4	4	4
64	4	4	4	4	4	4

RAW DATA ON THE STUDY INFLUENCE OF RISK AND CONTRACT MANAGEMENT PRACTICES AND PROJECT MANAGER'S SKILLS ON PERFORMANCE OF CONSTRUCTION PROJECTS

RISK MANAGEMENT PRACTICES

Indicate the extent to which you agree or disagree with the following statements on risk management practices. Use a scale of 1-5 where 1=strongly disagree, 2=disagree, 3- neutral, 4= agree and 5= strongly agree.

Q1	Construction projects I have been involved in have a formal process for identification potential risks.
Q2	Regular assessments are conducted to evaluate the impact and likelihood of identified risks
Q3	Projects I have been involved in have a comprehensive risk responses plan
Q4	Resources are allocated for implementation of risk responses
Q5	Risk management strategies are regularly monitored and reviewed
Q6	Risk management strategies are adjusted should new risks occur during project implementation

Response ID	Q1. Process	Q2.Assess	Q3. Plan	Q4. Resources	Q5. Monitor	Q6. Adjust

	Q1. Process	Q2.Assess	Q3. Plan	Q4. Resources	Q5. Monitor	Q6. Adjust	Total Averages
**Mean**	**4.11**	**4.08**	**4.02**	**3.86**	**4.31**	**4.13**	**4.08**
**S.D**	**0.31**	**0.32**	**0.28**	**0.35**	**0.47**	**0.33**	**0.35**
**Standard error**	**0.039**	**0.040**	**0.035**	**0.044**	**0.058**	**0.042**	**0.04**

RAW DATA ON THE STUDY INFLUENCE OF RISK AND CONTRACT MANAGEMENT PRACTICES AND PROJECT MANAGER'S SKILLS ON PERFORMANCE OF CONSTRUCTION PROJECTS

CONTRACT MANAGEMENT PRACTICES

Indicate the extent to which you agree or disagree with the following statements on contract management practices. Use a scale of 1-5 where 1=strongly disagree, 2=disagree, 3- neutral, 4= agree and 5= strongly agree.

Q1	Projects I have been involved in have a well-defined contract management procedure
Q2	Regular assessments are conducted to evaluate the impact and likelihood of identified risks
Q3	Conditions of contract are reviewed, negotiated and agreed upon by parties before signing
Q4	Parties' adherence to their contractual obligations is monitored regularly during project lifecycle
Q5	Disputes, conflicts and disagreements that arise are resolved efficiently and effectively

Response ID	Q1. CM Procedures	Q2.Contract review and negotiations	Q3. Monit	Q4. Dispute resolutions
1	5	5	5	4
2	4	4	4	4
3	4	4	4	4
4	5	4	4	4
5	4	4	4	4
6	4	4	4	4
7	4	4	4	3
8	4	4	4	4
9	5	5	5	4
10	4	4	4	4
11	4	4	4	4
12	4	4	4	4
13	5	5	5	5
14	4	4	4	4
15	4	4	4	4
16	4	4	4	4
17	4	4	4	4
18	5	5	4	4
19	4	4	4	4
20	4	4	4	3
21	4	4	4	4
22	5	5	5	4
23	4	4	4	4
24	4	4	4	4
25	4	4	4	4
26	4	4	4	4
27	5	4	4	4
28	4	4	4	4
29	4	4	4	4
30	4	4	4	4
31	4	4	4	4
32	4	4	4	4
33	4	4	4	4
34	4	4	4	4
35	4	4	4	4
36	5	5	5	4

RAW DATA ON THE STUDY INFLUENCE OF RISK AND CONTRACT MANAGEMENT PRACTICES AND PROJECT MANAGER'S SKILLS ON PERFORMANCE OF CONSTRUCTION PROJECTS

CONTRACT MANAGEMENT PRACTICES

Indicate the extent to which you agree or disagree with the following statements on contract management practices. Use a scale of 1-5 where 1=strongly disagree, 2=disagree, 3- neutral, 4= agree and 5= strongly agree.

Q1 Projects I have been involved in have a well-defined contract management procedure

Q2 Regular assessments are conducted to evaluate the impact and likelihood of identified risks

Q3 Conditions of contract are reviewed, negotiated and agreed upon by parties before signing

Q4 Parties' adherence to their contractual obligations is monitored regularly during project lifecycle

Q5 Disputes, conflicts and disagreements that arise are resolved efficiently and effectively

Response ID	Q1. CM Procedures	Q2.Contract review and negotiations	Q3. Monit	Q4. Dispute resolutions
37	4	4	4	4
38	4	4	4	4
39	4	4	4	4
40	4	4	4	4
41	4	4	4	4
42	4	4	4	4
43	4	4	4	4
44	4	4	4	4
45	4	4	4	4
46	4	4	4	4
47	4	4	4	4
48	4	4	4	4
49	4	4	4	4
50	4	4	4	4
51	4	4	4	4
52	5	5	5	5
53	4	4	4	4
54	4	4	4	4
55	4	4	4	4
56	5	5	5	4
57	4	4	4	4
58	4	4	4	4
59	4	4	4	3
60	4	4	4	4
61	4	4	4	4
62	4	4	4	4
63	4	4	4	4
64	4	4	4	4

RAW DATA ON THE STUDY INFLUENCE OF RISK AND CONTRACT MANAGEMENT PRACTICES AND PROJECT MANAGER'S SKILLS ON PERFORMANCE OF CONSTRUCTION PROJECTS

CONTRACT MANAGEMENT PRACTICES

Indicate the extent to which you agree or disagree with the following statements on contract management practices. Use a scale of 1-5 where 1=strongly disagree, 2=disagree, 3- neutral, 4= agree and 5= strongly agree.

Q1	Projects I have been involved in have a well-defined contract management procedure
Q2	Regular assessments are conducted to evaluate the impact and likelihood of identified risks
Q3	Conditions of contract are reviewed, negotiated and agreed upon by parties before signing
Q4	Parties' adherence to their contractual obligations is monitored regularly during project lifecycle
Q5	Disputes, conflicts and disagreements that arise are resolved efficiently and effectively

Response ID	Q1. CM Procedures	Q2.Contract review and negotiations	Q3. Monit	Q4. Dispute resolutions	Total Averages
**Mean**	**4.16**	**4.13**	**4.11**	**3.98**	**4.09**
**S.D**	**0.37**	**0.33**	**0.31**	**0.28**	**0.32**
**Standard error**	**0.046**	**0.042**	**0.039**	**0.035**	**0.04**

RAW DATA ON THE STUDY INFLUENCE OF RISK AND CONTRACT MANAGEMENT PRACTICES AND PROJECT MANAGER'S SKILLS ON PERFORMANCE OF CONSTRUCTION PROJECTS

PROJECT MANAGER'S SKILLS

Indicate the extent to which the following statements on project manager's skills. Use a scale of 1-5 where 1=strongly disagree, 2=disagree, 3- neutral, 4= agree and 5= strongly agree

Q1	Communication skills is an important skill for a construction project manager
Q2	Leadership skills is an important skill for a construction project manager
Q3	Problem solving skills is an important skill for a construction project manager
Q4	Risk management skills is an important skill for a construction project manager
Q5	Technical knowledge is an important skill for a construction project manager
Q6	Decision making skills is an important skill for a construction project manager

Response ID	Q1. Communication skills	Q2.Leadership Skills	Q3. Problem Solving Skills	Q4. Risk Management Skills	Q5. Technical Skills	6. Decision Making Skills
1	5	5	5	4	4	5
2	5	5	4	4	4	5
3	5	4	4	4	4	4
4	5	5	5	5	4	5
5	5	5	4	4	4	5
6	4	4	4	4	4	4
7	5	5	4	4	4	5
8	5	5	5	5	4	5
9	5	4	4	4	4	4
10	5	5	4	4	4	5
11	5	5	5	5	4	5
12	5	4	4	4	4	4
13	4	4	4	4	4	4
14	5	5	5	5	4	5
15	5	5	4	4	4	5
16	5	5	4	4	4	5
17	5	5	5	5	5	5
18	5	5	4	4	4	5
19	5	4	4	4	4	4
20	4	4	4	4	3	4
21	5	5	5	5	4	5
22	5	5	5	5	5	5
23	5	4	4	4	4	4
24	5	5	4	4	4	5
25	5	5	5	5	4	5
26	4	4	4	4	4	4
27	5	5	4	4	4	5
28	5	5	5	4	4	5
29	5	5	4	4	4	5
30	5	4	4	4	4	4
31	5	5	4	4	4	5
32	5	5	5	5	4	5
33	5	5	4	4	4	5
34	5	5	5	5	5	5
35	5	5	4	4	4	5
36	5	5	5	5	4	5

RAW DATA ON THE STUDY INFLUENCE OF RISK AND CONTRACT MANAGEMENT PRACTICES AND PROJECT MANAGER'S SKILLS ON PERFORMANCE OF CONSTRUCTION PROJECTS

PROJECT MANAGER'S SKILLS

Indicate the extent to which the following statements on project manager's skills. Use a scale of 1-5 where 1=strongly disagree, 2=disagree, 3- neutral, 4= agree and 5= strongly agree

Q1 Communication skills is an important skill for a construction project manager

Q2 Leadership skills is an important skill for a construction project manager

Q3 Problem solving skills is an important skill for a construction project manager

Q4 Risk management skills is an important skill for a construction project manager

Q5 Technical knowledge is an important skill for a construction project manager

Q6 Decision making skills is an important skill for a construction project manager

Response ID	Q1. Communication skills	Q2.Leadership Skills	Q3. Problem Solving Skills	Q4. Risk Management Skills	Q5. Technical Skills	6. Decision Making Skills
37	5	5	4	4	4	5
38	5	4	4	4	4	4
39	5	5	5	5	5	5
40	5	5	4	4	4	5
41	5	5	4	4	4	5
42	5	5	5	5	5	5
43	4	4	4	4	4	4
44	5	5	4	4	4	5
45	5	5	5	5	4	5
46	5	5	4	4	4	5
47	5	5	5	5	4	5
48	5	5	5	5	5	5
49	5	5	4	4	4	5
50	5	5	4	4	4	5
51	5	5	5	5	5	5
52	5	5	5	5	4	5
53	5	5	4	4	4	5
54	4	4	4	4	4	4
55	5	5	4	4	4	5
56	5	5	5	5	4	5
57	5	5	4	4	4	5
58	5	5	4	4	4	5
59	5	5	5	5	4	5
60	5	4	4	4	4	4
61	5	5	5	5	4	5
62	5	5	4	4	4	5
63	5	5	4	4	4	5
64	5	5	5	5	4	5

RAW DATA ON THE STUDY INFLUENCE OF RISK AND CONTRACT MANAGEMENT PRACTICES AND PROJECT MANAGER'S SKILLS ON PERFORMANCE OF CONSTRUCTION PROJECTS

PROJECT MANAGER'S SKILLS

Indicate the extent to which the following statements on project manager’s skills. Use a scale of 1-5 where
1=strongly disagree, 2=disagree, 3- neutral, 4= agree and 5= strongly agree

Q1	Communication skills is an important skill for a construction project manager
Q2	Leadership skills is an important skill for a construction project manager
Q3	Problem solving skills is an important skill for a construction project manager
Q4	Risk management skills is an important skill for a construction project manager
Q5	Technical knowledge is an important skill for a construction project manager
Q6	Decision making skills is an important skill for a construction project manager

Response ID	Q1. Communication skills	Q2.Leadership Skills	Q3. Problem Solving Skills	Q4. Risk Management Skills	Q5. Technical Skills	6. Decision Making Skills	Total Averages
**Mean**	**4.91**	**4.78**	**4.38**	**4.34**	**4.09**	**4.78**	**4.55**
**S.D**	**0.29**	**0.42**	**0.49**	**0.48**	**0.34**	**0.42**	**0.41**
**Standard error**	**0.037**	**0.052**	**0.061**	**0.060**	**0.043**	**0.052**	**0.05**

RAW DATA ON THE STUDY INFLUENCE OF RISK AND CONTRACT MANAGEMENT PRACTICES AND PROJECT MANAGER'S SKILLS ON PERFORMANCE OF CONSTRUCTION PROJECTS

PROJECT PERFORMANCE MEASURES

Please rate the following project performance measures. Use a scale of 1-5 where 1=strongly disagree, 2=disagree, 3- neutral, 4= agree and 5= strongly agree.

Q1	Quality
Q2	Time
Q3	Budget
Q4	Clients' satisfaction
Q5	Safety
Q6	Environmental

Response ID	Q1. Quality	Q2.Time	Q3. Budget	Q4. Client's satisfaction	Q5. Safety	6. Environmental
1	5	4	4	5	5	4
2	4	4	4	5	4	4
3	4	4	4	4	4	4
4	5	5	5	5	5	4
5	4	4	4	4	4	4
6	4	4	4	4	4	4
7	5	4	4	5	4	3
8	4	4	4	5	5	4
9	5	5	5	5	5	4
10	4	4	4	4	4	4
11	4	4	4	5	4	4
12	4	4	4	4	4	4
13	4	4	4	4	4	4
14	4	4	4	4	4	4
15	5	5	5	5	5	4
16	4	4	4	4	4	4
17	4	4	4	4	4	4
18	5	5	5	5	5	4
19	4	4	4	4	4	4
20	4	4	4	4	4	3
21	4	4	4	5	4	4
22	5	5	5	5	5	4
23	4	4	4	4	4	4
24	4	4	4	4	4	4
25	4	4	4	4	4	4
26	4	4	4	4	4	4
27	4	4	4	5	4	4
28	4	4	4	4	4	4
29	5	5	5	5	5	4
30	4	4	4	4	4	4
31	4	4	4	4	4	4
32	5	5	5	5	5	4
33	4	4	4	4	4	4
34	4	4	4	4	4	4
35	4	4	4	4	4	4

RAW DATA ON THE STUDY INFLUENCE OF RISK AND CONTRACT MANAGEMENT PRACTICES AND PROJECT MANAGER'S SKILLS ON PERFORMANCE OF CONSTRUCTION PROJECTS

PROJECT PERFORMANCE MEASURES

Please rate the following project performance measures. Use a scale of 1-5 where 1=strongly disagree, 2=disagree, 3- neutral, 4= agree and 5= strongly agree.

Q1 Quality

Q2 Time

Q3 Budget

Q4 Clients’ satisfaction

Q5 Safety

Q6 Environmental

Response ID	Q1. Quality	Q2.Time	Q3. Budget	Q4. Client's satisfaction	Q5. Safety	6. Environmental
36	5	5	5	5	5	4
37	4	4	4	4	4	4
38	4	4	4	4	4	4
39	4	4	4	5	4	4
40	4	4	4	4	4	4
41	4	4	4	4	4	4
42	4	4	4	4	4	4
43	4	4	4	4	4	4
44	4	4	4	5	4	4
45	4	4	4	4	4	4
46	4	4	4	4	4	4
47	4	4	4	4	4	4
48	5	5	5	5	5	4
49	4	4	4	4	4	4
50	4	4	4	4	4	4
51	4	4	4	4	4	4
52	5	5	5	5	5	4
53	4	4	4	4	4	4
54	4	4	4	4	4	4
55	4	4	4	4	4	4
56	5	5	5	5	5	4
57	4	4	4	4	4	4
58	4	4	4	4	4	4
59	4	4	4	4	4	4
60	4	4	4	4	4	4
61	4	4	4	5	4	4
62	4	4	4	4	4	4
63	4	4	4	4	4	4
64	4	4	4	4	4	4

RAW DATA ON THE STUDY INFLUENCE OF RISK AND CONTRACT MANAGEMENT PRACTICES AND PROJECT MANAGER'S SKILLS ON PERFORMANCE OF CONSTRUCTION PROJECTS

PROJECT PERFORMANCE MEASURES

Please rate the following project performance measures. Use a scale of 1-5 where 1=strongly disagree, 2=disagree, 3- neutral, 4= agree and 5= strongly agree.

Q1	Quality
Q2	Time
Q3	Budget
Q4	Clients' satisfaction
Q5	Safety
Q6	Environmental

Response ID	Q1. Quality	Q2.Time	Q3. Budget	Q4. Client's satisfaction	Q5. Safety	6. Environmental	Total Average
**Mean**	**4.20**	**4.17**	**4.17**	**4.33**	**4.20**	**3.97**	**4.17**
**S.D**	**0.41**	**0.38**	**0.38**	**0.47**	**0.41**	**0.18**	**0.37**
**Standard error**	**0.051**	**0.048**	**0.048**	**0.059**	**0.051**	**0.022**	**0.05**

RAW DATA ON THE STUDY INFLUENCE OF RISK AND CONTRACT MANAGEMENT PRACTICES AND PROJECT MANAGER'S SKILLS ON PERFORMANCE OF CONSTRUCTION PROJECTS

FACTORS INFLUENCING PROJECT PERFORMANCE

Indicate the extent to which the following factors affect project performance. Use a scale of 1-5 where 1=No extent, 2=little extent, 3- moderate extent, 4= great extent and 5= very great extent

Q1 To what extent do effective risk management practices influence performance of construction projects

Q2 To what extent do effective contract management practices contribute to overall performance of construction projects

Q3 To what extent do relevant project manager's skills contribute to overall performance of construction projects

Response ID	Factor 1. RM Practices	Factor 2: CM Practices	Factor 3: PM Skills
1	5	4	5
2	4	4	5
3	4	4	4
4	5	5	5
5	4	4	4
6	4	4	4
7	4	4	4
8	4	4	5
9	5	5	5
10	4	4	4
11	4	4	5
12	4	4	4
13	4	4	4
14	4	4	4
15	5	5	5
16	4	4	4
17	4	4	4
18	5	5	5
19	4	4	4
20	4	4	4
21	4	4	5
22	5	5	5
23	4	4	4
24	4	4	4
25	4	4	4
26	4	4	4
27	4	4	5
28	4	4	4
29	5	5	5
30	4	4	4
31	4	4	4

RAW DATA ON THE STUDY INFLUENCE OF RISK AND CONTRACT MANAGEMENT PRACTICES AND PROJECT MANAGER'S SKILLS ON PERFORMANCE OF CONSTRUCTION PROJECTS

FACTORS INFLUENCING PROJECT PERFORMANCE

Indicate the extent to which the following factors affect project performance. Use a scale of 1-5 where 1=No extent, 2=little extent, 3- moderate extent, 4= great extent and 5= very great extent

Q1 To what extent do effective risk management practices influence performance of construction projects

Q2 To what extent do effective contract management practices contribute to overall performance of construction projects

Q3 To what extent do relevant project manager's skills contribute to overall performance of construction projects

Response ID	Factor 1. RM Practices	Factor 2: CM Practices	Factor 3: PM Skills
32	5	5	5
33	4	4	4
34	4	4	4
35	4	4	4
36	5	5	5
37	4	4	4
38	4	4	4
39	4	4	5
40	4	4	4
41	4	4	4
42	4	4	4
43	4	4	4
44	4	4	5
45	4	4	4
46	4	4	4
47	4	4	4
48	5	5	5
49	4	4	4
50	4	4	4
51	4	4	4
52	5	5	5
53	4	4	4
54	4	4	4
55	4	4	4
56	5	5	5
57	4	4	4
58	4	4	4
59	4	4	4
60	4	4	4
61	4	4	5
62	4	4	4

RAW DATA ON THE STUDY INFLUENCE OF RISK AND CONTRACT MANAGEMENT PRACTICES AND PROJECT MANAGER'S SKILLS ON PERFORMANCE OF CONSTRUCTION PROJECTS

FACTORS INFLUENCING PROJECT PERFORMANCE

Indicate the extent to which the following factors affect project performance. Use a scale of 1-5 where 1=No extent, 2=little extent, 3- moderate extent, 4= great extent and 5= very great extent

Q1 To what extent do effective risk management practices influence performance of construction projects

Q2 To what extent do effective contract management practices contribute to overall performance of construction projects

Q3 To what extent do relevant project manager's skills contribute to overall performance of construction projects

Response ID	Factor 1. RM Practices	Factor 2: CM Practices	Factor 3: PM Skills
63	4	4	4
64	4	4	4
**Mean**	**4.19**	**4.17**	**4.31**
**S.D**	**0.39**	**0.38**	**0.47**
**Standard erro**	**0.049**	**0.048**	**0.058**
